# Characterization of a new L-carnosine synthase mined from deep-sea sediment metagenome

**DOI:** 10.1186/s12934-022-01854-w

**Published:** 2022-06-27

**Authors:** Jiajia She, Lihong Fu, Xiaowei Zheng, Jing Li, Limin Wang, Bo Yu, Jiansong Ju

**Affiliations:** 1 College of Life Science, Hebei Normal University, Shijiazhuang, 050024 China; 2 Institute of Microbiology, Chinese Academy of Sciences, Beijing, 100101 China; 3 Hebei Collaborative Innovation Center for Eco-Environment, Shijiazhuang, 050024 China

**Keywords:** L-carnosine, β-aminopeptidase, Gene mining, Deep-sea metagenome

## Abstract

L-Carnosine is a natural biologically active dipeptide with critical physiological functions, such as antioxidant, antiglycation, and cytoplasmic buffering properties. Direct enzymatic synthesis is a promising way for L-carnosine production. In this study, a new aminopeptidase (gene_236976) with synthetic activity toward L-carnosine was identified by a metagenome mining approach from deep-sea sediment and functionally expressed in *Escherichia coli*. The enzyme shared a low identity of 14.3% with reported L-carnosine dipeptidase (*Sm*PepD) from *Serratia marcescens*. β-Alanine methyl ester was proven to be the best substrate for the synthesis, and no ATP was needed for the enzymatic reaction. The enzyme activity was increased by structure-guided rational design. Only the mutant of G310 site gave positive results, and G310A mutant showed the best performance among the site-direct saturation mutagenesis, indicating that the additional CH_3_ group of mutant G310A was the main factor affecting the enzymatic activity. The engineered enzyme produced about 10 mM L-carnosine was produced from substrates of 50 mM β-alanine methyl ester and 50 mM L-histidine, under a tentatively optimized condition. This study enriched the enzyme resources for developing the microbial synthesis process of L-carnosine production.

**Supplementary Information:**

The online version contains supplementary material available at 10.1186/s12934-022-01854-w.

## Introduction

L-Carnosine (β-alanyl-L-histidine) is a dipeptide of the amino acids β-alanine and histidine, which have long been reported to be highly concentrated in skeletal muscle and the central nervous system of vertebrates [1]. L-Carnosine has many critical physiological functions in vivo, including anti-oxidation, anti-glycosylation, cytoplasmic buffering, and free hydroxyl radical elimination. Therefore, L-carnosine has attracted attention as a bioactive compound and is widely used in medicine, cosmetics, food additives, and other fields [2]. Chemical synthesis of L-carnosine has been extensively reported and become the currently commercial way for L-carnosine production. The chemical production process requires many complicated reactions with protected amino acids and highly toxic reagents. Moreover, the severe reaction conditions and high-energy consumption also make this process environmentally unfriendly, which other methods should replace.

Enzymatic synthesis of dipeptides has become a popular research topic because of its excellent selectivity and environment-friendly manner [2, 3]. Carnosine has been considered to be synthesized by the called carnosine synthase from β-alanine and histidine in many tissues. Mg^2+^ and ATP are required to synthesize carnosine in addition to the constituent amino acids [4, 5]. Drozak et al. [6] first molecularly identified the carnosine synthase from chicken pectoral muscle. The enzyme is thought to be a homotetramer with a molecular mass of ∼43.0 kDa for the native enzyme. This breakthrough resolved and corrected the reaction properties that the chicken carnosine synthase stoichiometrically forms ADP + phosphate, rather than AMP + pyrophosphate as suggested in previous studies. Interestingly, Tsubone et al. [7] reported a non-ATP-dependent imidazole dipeptide synthase capable of synthesizing carnosine, which was purified from Japanese eel. Besides carnosine synthase, β-aminopeptidases exhibit both hydrolytic and aminolytic (peptide bond formation) activities and have only been reported in bacteria [8, 9]. Many aminopeptidases have been demonstrated, and this new peptidase family is rapidly expanding. The dinuclear zinc aminopeptidase (PepV) from *Lactobacillus delbrueckii* is one of the first characterized bacterial enzymes that can hydrolyze L-carnosine [10]. Similarly, the aminopeptidase (DmpA) from *Ochrobactrum anthropi*, β-alanyl-Xaa dipeptidase (BapA, BapF) from *Pseudomonas* sp. show high specificity towards carnosine degradation [11, 12]. Aminoacyl-histidine dipeptidases (Xaa-His dipeptidase, PepD) have also been cloned from *Vibrio alginolyticus* and *Porphyromonas gingivalis* [13, 14].

Based on the revealed enzymes, microbial synthesis of L-carnosine has been explored by using non-protected β-alanine or its activated derivatives and L-histidine as substrates. Kino [15] engineered an L-amino acid ligase (YwfE) from *Bacillus subtilis* to synthesize L-carnosine in an ATP-dependent manner. The best mutant obtained a high molar yield of 91.4% at a substrate loading of 12.5 mM. Since the required equal molar amount of ATP made the process impractical, the non-ATP-dependent synthesis processes were also explored. The yeast cell surface-displayed carnosinase CN1 was employed to synthesize L-carnosine, while a low molar yield of 4.5% was obtained with 500 mM β-alanine and 100 mM L-histidine [16].

The β-peptidyl aminopeptidase (BapA) from *Sphingosinicella microcystinivorans* Y2 has been reported to catalyze the carnosine synthesis with β-alanyl-*para*-nitroaniline as the acyl donor [9]. Using aminopeptidase (DmpA) from *Ochrobactrum anthropi*, a simple enzymatic procedure for L-carnosine synthesis was established by using the recyclable whole-cell biocatalyst [17]. The high yields of up to 71% were obtained from the activated derivatives of β-alanine, β-alaninamide, and 3.7 g/L L-carnosine was accumulated in a fed-batch process. Recently, a genome mining approach identified a highly active dipeptidase (*Sm*PepD) from *Serratia marcescens* [2]. Under the optimized reaction conditions, *Sm*PepD could efficiently catalyze the coupling of β-alanine and L-histidine, and the productivity of L-carnosine reached 60.3 g/L/d by adding 5 g lyophilized cell free extract (750 U/g). A kilogram scale production process with nanofiltration purification steps was also proposed.

Although some bioconversion processes were reported, the information on the enzymes that catalyze the formation of L-carnosine and related dipeptides still remains deficient. In this paper, a novel aminopeptidase with low identities with currently reported dipeptidases was successfully mined from the deep-sea sediment metagenome sequences. The functionality of the enzyme was confirmed and the activity was also improved by a structure-guided rational design.

## Materials and methods

### Strains and cultivation conditions

The DNA sequence of L-carnosine synthase gene used in this study was first synthesized and then cloned into the expression vector of pET-26b with restriction sites of *Nco*I and *Xho*I to get the recombinant plasmid pET26b-WT_Car_. The L-carnosine synthase genes were expressed in strain *Escherichia coli* BL21(DE3). The strains were cultivated in Luria–Bertani (LB) medium with 40 μg/mL kanamycin. The recombinant strains were inoculated into LB medium with antibiotic and cultivated overnight at 37 °C, 200 rpm. Then two milliliters of the overnight culture were inoculated into 200 mL of fresh LB medium with 40 μg/mL kanamycin. When the OD_600_ value reached around 0.6 ~ 0.8, 0.02 mM isopropyl-β-d-1-thiogalactopyranoside (IPTG) was added to promote protein expression under 16 °C overnight.

### Homology modeling of aminopeptidase

The amino acid sequence of L-aminopeptidase was retrieved from a deep-sea sediment metagenome sequence (NCBI accession number SRR8302209). Sequence similarity search was performed with the BLAST program within the protein data bank (PDB) [18–20]. Coordinates of the X-ray crystal structure (PDB code: 1B65), which holds a high amino acid sequence similarity of 45.1% (BLASTP suite) with our L-aminopeptidase, were used as the template [21]. Sequence alignment was performed, and the 3D_model of L-aminopeptidase was generated using Modeller 9v21 [22]. The tertiary structural model of L-aminopeptidase and its mutants was built automatically by Swiss-Model Automatic Modelling Mode [23].

### Docking of substrate into aminopeptidase

Superimposition was then carried out between the generated model and the X-ray crystal structure of β-aminopeptidase from *Sphingosinicella xenopeptidilytica* (PDB code: 3NFB). The substrate that existed in 3NFB was extracted to the corresponding active site of L-aminopeptidase and then modified to be L-carnosine synthase using PyMol v2.4.0. Residues whose atoms are within 3 Å of the substrate were selected to be examined in the virtual screening.

### Computer-aided saturation mutagenesis

In order to predict mutations with improved activity towards L-carnosine synthesis, computer-aided saturation mutagenesis was conducted for virtual screening. For this purpose, each residue located around 3 Å of the docked substrate mutated to the other types of amino acids, and the corresponding conformation of L-carnosine in the substrate binding pocket was predicted by AutoDock Vina [24]. Mutations were selected to be tested in experiments. Site-directed mutagenesis was performed by PCR [25] by using pET26b-WT_Car_ as the template. The nucleotide sequences of primers used for mutagenesis are shown in Table 1.

**Table 1:** Primers used for site mutations in this study

primers	Sequence (5′-3′)
W133D-F	TGGCCGGCTGACgacCACGCTCCGGTTGTT
W133D-R	CGGAGCGTGgtcGTCAGCCGGCCAACCCTG
Y142N-F	GTTGTTGCTGAAACCaacGACGGTGGTCTG
Y142N-R	TCGTTCAGACCACCGTCgttGGTTTCAGC
Y142K-F	TTGTTGCTGAAACCaaaGACGGTGGTCTG
Y142K-R	TCGTTCAGACCACCGTCtttGGTTTCAGC
S86Q-F	AACGCTcagGGTGAAATGACCGGTACCACCTGGC
S86Q-R	GCCAGGTGGTACCGGTCATTTCACCctgAGCGTT
D218E-F	GTGCAACTACAACTGGgaaGGTGAACAGGACCTG
D218E-R	CAGGTCCTGTTCACCttcCCAGTTGTAGTTGCAC
G310A-F	CTCTTCTGACgctTCTGGTGACATCTTCCTGGC
G310A-R	GCCAGGAAGATGTCACCAGAagcGTCAGAAGAG
S86NG310A-F	GAACGCTaacGGTGAAATGACCGGTACCACC
S86NG310A-R	GGTGGTACCGGTCATTTCACCgttAGCGTTC
R270GG310A-F	CAAACCGACCggtGACGGTTCTATCATCATC
R270GG310A-R	GATGATGATAGAACCGTCaccGGTCGGTTTG

The mutated plasmids were constructed using high-fidelity Q5 DNA polymerase. The resultant plasmids were digested with the restriction enzyme *Dpn*I at 37 °C for 2 h. The mutants were designated as W133D, Y142N, Y142K, S86Q, D218E, G310A for single-site mutation as well as S86N/G310A and R270G/G310A for double site mutation, respectively. The saturation mutation at position of G310 was performed as the same procedures and the primers used were shown in Table 2. All the mutations were confirmed by gene sequencing.

**Table 2:** The primers used for saturation mutation at position G310 in this study

Primers	Sequences (5′-3′)
G310K-F	CTCTTCTGACaaaTCTGGTGACATCTTCCTGGC
G310K-R	CCAGGAAGATGTCACCAGAtttGTCAGAAGAG
G310P-F	CTCTTCTGACccgTCTGGTGACATCTTCCTGGC
G310P-R	GCCAGGAAGATGTCACCAGAcggGTCAGAAGAG
G310R-F	CTCTTCTGACcgtTCTGGTGACATCTTCCTGGC
G310R-R	GCCAGGAAGATGTCACCAGAacgGTCAGAAGAG
G310T-F	CTCTTCTGACaccTCTGGTGACATCTTCCTGGC
G310T-R	GCCAGGAAGATGTCACCAGAggtGTCAGAAGAG
G310D-F	CTCTTCTGACgacTCTGGTGACATCTTCCTGGC
G310D-R	GCCAGGAAGATGTCACCAGAgtcGTCAGAAGAG
G310S-F	CTCTTCTGACtctTCTGGTGACATCTTCCTGGC
G310S-R	GCCAGGAAGATGTCACCAGAagaGTCAGAAGAG
G310I-F	CTCTTCTGACatcTCTGGTGACATCTTCCTGGC
G310I-R	GCCAGGAAGATGTCACCAGAgatGTCAGAAGAG
G310V-F	CTCTTCTGACgttTCTGGTGACATCTTCCTGGC
G310V-R	GCCAGGAAGATGTCACCAGAaacGTCAGAAGAG
G310L-F	CTCTTCTGACctgTCTGGTGACATCTTCCTGGC
G310L-R	GCCAGGAAGATGTCACCAGAcagGTCAGAAGAG
G310H-F	CTCTTCTGACcacTCTGGTGACATCTTCCTGGC
G310H-R	GCCAGGAAGATGTCACCAGAgtgGTCAGAAGAG
G310Q-F	CTCTTCTGACcagTCTGGTGACATCTTCCTGGC
G310Q-R	GCCAGGAAGATGTCACCAGActgGTCAGAAGAG
G310F-F	CTCTTCTGACttcTCTGGTGACATCTTCCTGGC
G310F-R	GCCAGGAAGATGTCACCAGAgaaGTCAGAAGAG
G310N-F	CTCTTCTGACaacTCTGGTGACATCTTCCTGGC
G310N-R	GCCAGGAAGATGTCACCAGAgttGTCAGAAGAG
G310E-F	CTCTTCTGACgaaTCTGGTGACATCTTCCTGGC
G310E-R	GCCAGGAAGATGTCACCAGAttcGTCAGAAGAG
G310M-F	CTCTTCTGACatgTCTGGTGACATCTTCCTGGC
G310M-R	GCCAGGAAGATGTCACCAGAcatGTCAGAAGAG
G310W-F	CTCTTCTGACtggTCTGGTGACATCTTCCTGGC
G310W-R	GCCAGGAAGATGTCACCAGAccaGTCAGAAGAG
G310C-F	CTCTTCTGACtgcTCTGGTGACATCTTCCTGGC
G310C-R	GCCAGGAAGATGTCACCAGAgcaGTCAGAAGAG
G310Y-F	CTCTTCTGACtacTCTGGTGACATCTTCCTGGC
G310Y-R	GCCAGGAAGATGTCACCAGAgtaGTCAGAAGAG

### Enzyme activity assay

For selecting the best enzyme, the enzymatic activities were detected by whole cell reaction. The induced cells were centrifuged at 5000 rpm for 5 min, and the bacterial precipitation was washed twice with Na_2_CO_3_/NaHCO_3_ buffer (pH 10.0) and re-suspended in the same buffer to the OD_600_ value of 20. The substrate concentration of β-alanine-amide was set at 10 mM and 50 mM for L-histidine, as indicated by the previous report [17]. The reaction was conducted at 30 °C, 200 rpm, and stopped by adding 0.3 M HCl. The samples were measured by high-performance liquid chromatography (HPLC). Two additional β-alanine donor substrates were also tested with the same concentrations of β-alanine-amide, including β-alanine methyl ester hydrochloride (β-AlaOMe) and β-alanine ethyl ester hydrochloride (β-AlaOEt). The specific enzymatic activity measured with β-alanine-amide (H-β-Ala-NH_2_HCl) was defined as 100%.

The activities of all variants were measured using purified enzymes. In brief, all the his_6_-tagged enzymes were purified by traditional Ni-Agarose according to the protocol of the commercial kit. The enzymatic reaction system included 10 mM β-AlaOMe, 50 mM L-histidine, 74 μg/mL enzymes in a Na_2_CO_3_/NaHCO_3_ buffer (pH 10.0). The reaction was conducted in a water bath at 30 °C, stopped by adding 50 μL of 6 M HCl, and subjected to HPLC analyses.

### Analytical methods

The enzyme activity was determined by HPLC with reversed phase chromatography using NH_2_ column (200 mm × 4.6 mm, 5 μm) as the stationary phase, and the mobile phase were 44% acetonitrile and 56% 40 mM dipotassium hydrogen phosphate solution (pH 6.3, adjusted with phosphoric acid). The L-carnosine product determination parameters are as follows: flow rate of 1.0 mL/min, ultraviolet detection wavelength of 210 nm, column temperature of 25 °C, injection volume of 10 μL. The analytical method was verified with authentic standard L-carnosine. The concentrations in samples were quantitatively determined with the calibration curve using linear regression.

The product was further analyzed by LC–MS with a C18 column (250 mm × 4.6 mm, 5 μm). The detection condition was as follows: the mobile phase included 0.2% (v/v) formic acid solution (65%) and acetonitrile (35%), the flow rate was 0.5 mL/min, ultraviolet detection wavelength was set at 210 nm, the column temperature was 25 °C and the injection volume was 50 μL. LC–MS mass spectrometry was analyzed with electrospray ion source (ESI) and the method of negative ion detection. The scanning range was set from *m/z* 50 to 500. The interface temperature was 350 °C, and the desolvation line temperature was 250 °C with an atomizer flow of 1.5 L/min. The heating block temperature was set at 200 °C.

## Results

### The L-carnosine synthase gene mining from deep-sea sediment metagenome

Firstly, a local unique protein set was constructed from a deep-sea sediment metagenome (unpublished data). Briefly, our in-depth metagenomic sequencing reads (NCBI accession number SRR8302209) of the deep-sea sediment N6 (3,104 m) sampled from Northwest Indian Ocean, were adapter trimmed and removed for low-quality sequences using Btrim 0.3.0 (-w 20, -a 24, -l 80) [26], before being assembled into contigs using IDBA-UD V.1.1.1 (-maxk 250) [27]. Open reading frames (ORFs) were predicted from the resulting contigs using Prodigal V.2.6.3 [28], followed by clustered using CD-HIT at 60% identity and 80% alignment on the shorter amino acid sequence [29]. The amino acid sequence of L-carnosine synthase DmpA [17] was aligned against the unique protein set using diamond (V.0.9.10.111) with an E-value of 1e-3 [30]. Twenty-two putative homologous proteins were obtained after filtrating the matched results by ≥ 30% identity and ≥ 50% alignment. The 22 amino acid sequences, the phylogenetic tree and the figure of sequence alignment were all listed in the Additional file 1: Note S1, Additional file 2: Figures S1 and Additional file 3: Figures S2, respectively. Two gene sequences, including gene_1065070 with the highest identity of 57.9% and gene_236976 with the lowest identity of 44.8%, were firstly selected for codon optimization and synthesized for expression in *E. coli* BL21(DE3).

**Fig. 1:**
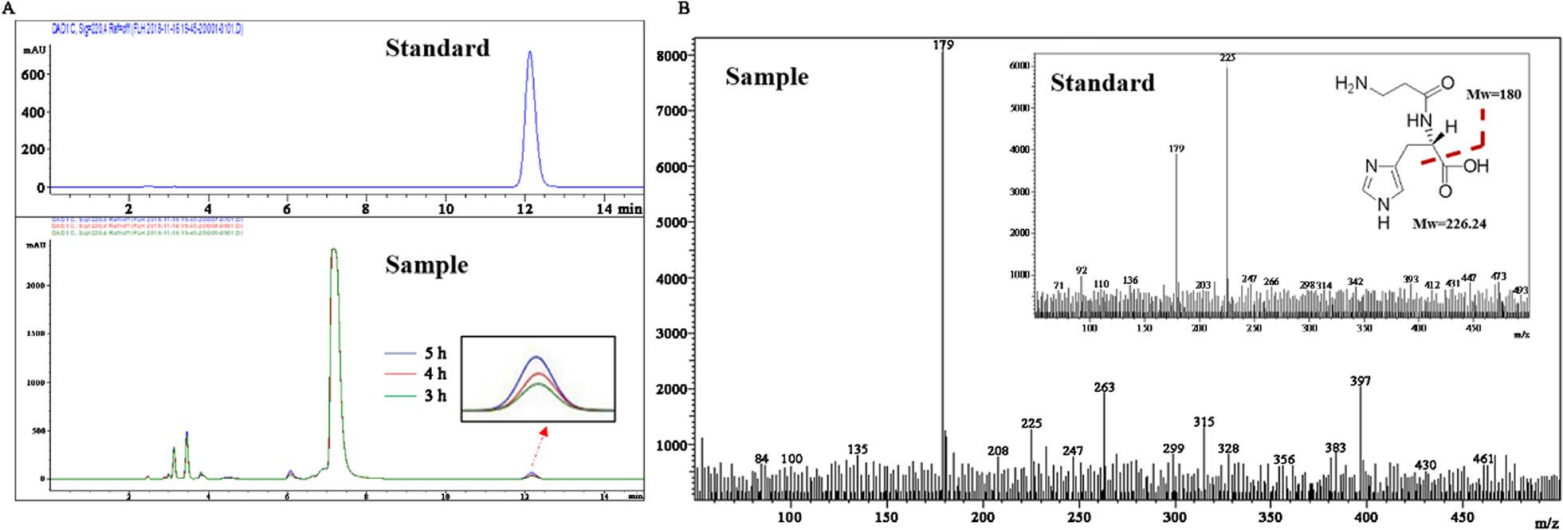
Chromatogram profile of produced L-carnosine. **a** HPLC chromatogram of the samples in different reaction time (3 h, 4 h, 5 h). The standard is HPLC chromatogram of L-carnosine standard. The arrow indicated the magnification of the product peaks in the samples. **b** LC–MS chromatogram profiles of L-carnosine standard and produced L-carnosine

The enzymatic activities were first measured by using β-alanine-amide and L-histidine as substrates. 0.5 mM ATP was also added in the whole-cell catalysis system to promote the reaction. However, ATP was later proven to have no contributory role in this reaction, and it was omitted in all subsequent experiments (data not shown). As shown in Fig. 1, only the gene_236976 product expressed the synthesis activity. The peak area of the product increased with the time and the peak was verified to be L-carnosine by LC–MS. The molecular weight of L-carnosine is 226, and the enzymatic product gave the molecular weight of 225 under the negative ion detection, which showed the same as the authentic standard L-carnosine. Additionally, the typical fragment ion peaks with the molecular weight of 179 were also identical. Thus, a new L-carnosine synthase gene was successfully mined from a deep-sea sediment metagenome in this study. The his_6_-tagged proteins of gene_236976 were purified by Ni-Agarose and shown in the Additional file 4: Figures S3. The enzyme could also hydrolysis L-carnosine, in which about 40% L-carnosine was degraded within 24 h with an initial concentration of 2.0 g/L. This result is consistent with previous reports, in which aminopeptidase catalyzes the reversible reaction [17].

### β-Alanine methyl ester served as the better substrate for L-carnosine synthesis

**Fig. 2:**
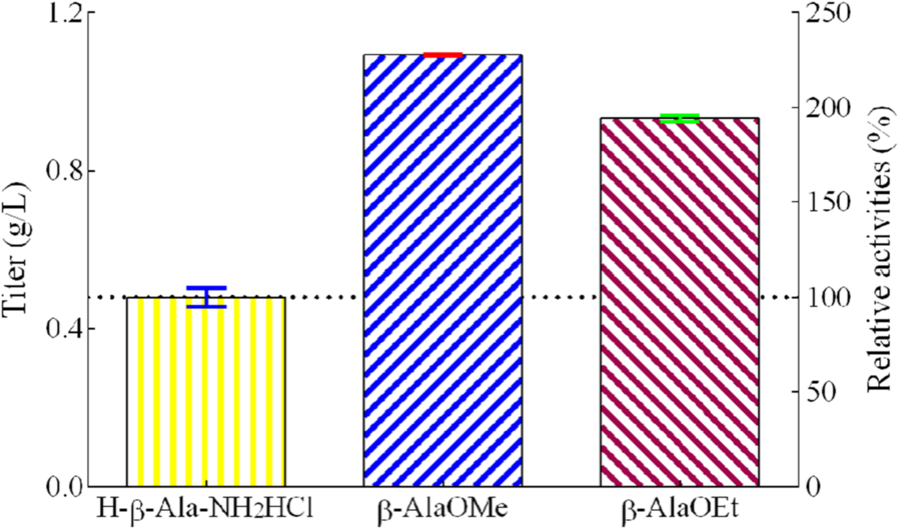
Effect of different as the acyl donors on the production of L-carnosine. The specific enzymatic activity measured from H-β-Ala-NH_2_HCl (β-alanine-amide) was defined as 100% (titer, 0.48 ± 0.02 g/L). β-AlaOMe, β-alanine methyl ester hydrochloride, and β-AlaOEt, β-alanine ethyl ester hydrochloride

The search for inexpensive raw materials is vital to reducing production cost of L-carnosine synthesis. Firstly, β-alanine was used as substrate with L-histidine and ATP to produce L-carnosine, while no product was obtained. Li et al. [2] reported using L-alanine ester to increase the production of L-alanyl-L-glutamine (Ala-Gln). In this study, β-alanine methyl ester hydrochloride (β-AlaOMe) and β-alanine ethyl ester hydrochloride (β-AlaOEt) were tested. As shown in **Fig. ****2**, an enzyme of gene_236976 showed activities with the two above substrates, much higher than the activity obtained with β-alanine-amide and L-histidine. β-AlaOMe was proven to be a better substrate since it expressed a 2.3-fold high activity (titer, 1.09 ± 0.00 g/L) compared to that with β-alanine-amide as substrate (titer, 0.48 ± 0.02 g/L). Even β-AlaOEt also showed around two-fold high activity (titer, 0.93 ± 0.01 g/L) than β-alanine-amide. As a primary conclusion, β-alanine ester could be the more suitable substrate for L-carnosine synthesis and showed a promising cheap feedstock for an industrial setting.

### Increase of the enzyme activity by structure-guide rational design

**Fig. 3:**
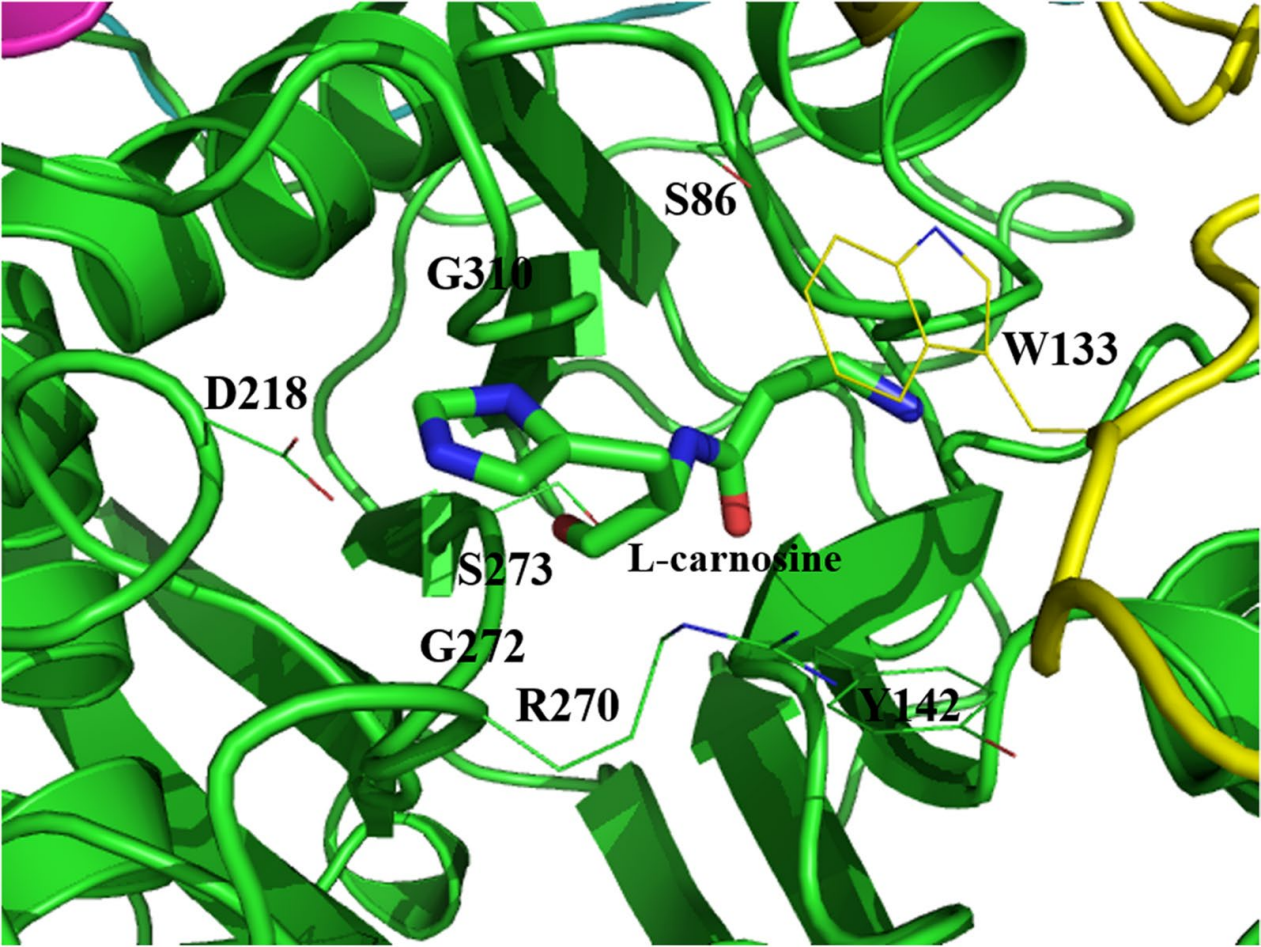
Substrate binding pocket of aminopeptidase generated by homology modeling and molecular docking. The 3D structure of L-aminopeptidase is shown in the mode of Cartoon with subunits given in different colors (green and yellow). The substrate is illustrated in sticks. Residues whose atoms are within 3 Å of the substrate are in lines

**Fig. 4:**
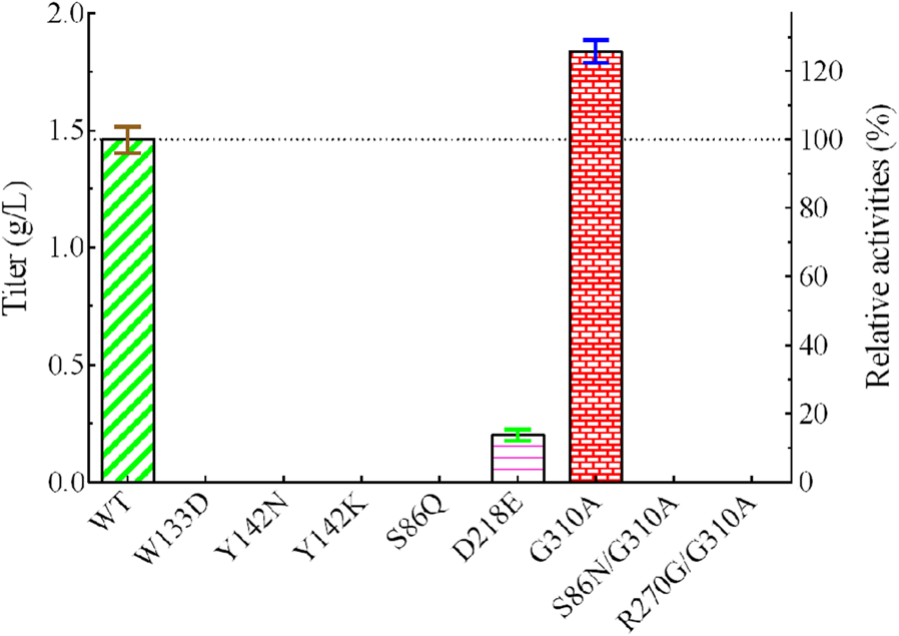
The production performance of L-carnosine by the wild-type and mutants. WT stands for wild-type, and mutants with the respective site-mutation are indicated. Each point represents the average of three data, where the error bars represent the standard deviation. The left Y-axis represents titer and the right Y-axis represents the relative activities. The activity data from that of wild-type (titer, 1.46 ± 0.06 g/L) was set as 100%

Based on the structures of L-aminopeptidase from *Ochrobactrum anthropic* (PDB code: 1B65) [21] and β-aminopeptidase from *S. xenopeptidilytica* (PDB code: 3NFB), homology modeling and molecular docking were performed to generate the substrate binding pocket. Residues whose atoms are within 3 Å of the substrate were selected, and those far away from the catalytic center were examined via computer-aided saturation mutagenesis (Fig. 3). Although residues of G272 and S273 are in the distance of 3 Å to the substrate, they are close to the catalytic center, which were not considered for mutagenesis. Mutations with which the conformation of L-carnosine was close to the one generated in the initial 3D model were selected to be verified by experiments. Based on the results of single-site mutagenesis, the enzyme activities of the respective mutants, including W133D, Y142N, Y142K, S86Q, D218E and G310A, were first experimentally investigated. As shown in Fig. 4, except for G310A and D218E, almost all other mutants were completely inactive, while the mutant D218E only retained 14% of its activity (titer, 0.20 ± 0.02 g/L), indicating that the enzyme activity is very susceptible to the binding pocket structure. Fortunately, the mutant G310A improved the enzyme activity by 25.7% (titer, 1.84 ± 0.05 g/L), as compared to the wild-type enzyme (titer, 1.46 ± 0.06 g/L). With the mutant G310A as the starting enzyme, double-site mutations of S86N/G310A and R270G/G310A were indicated by the second round of computer-aided saturation mutagenesis. However, these double-site mutants exhibited no activity towards L-carnosine synthesis in our experiments, indicating that the R270 site is also crucial to the enzyme activity (Fig. 4).

**Fig. 5:**
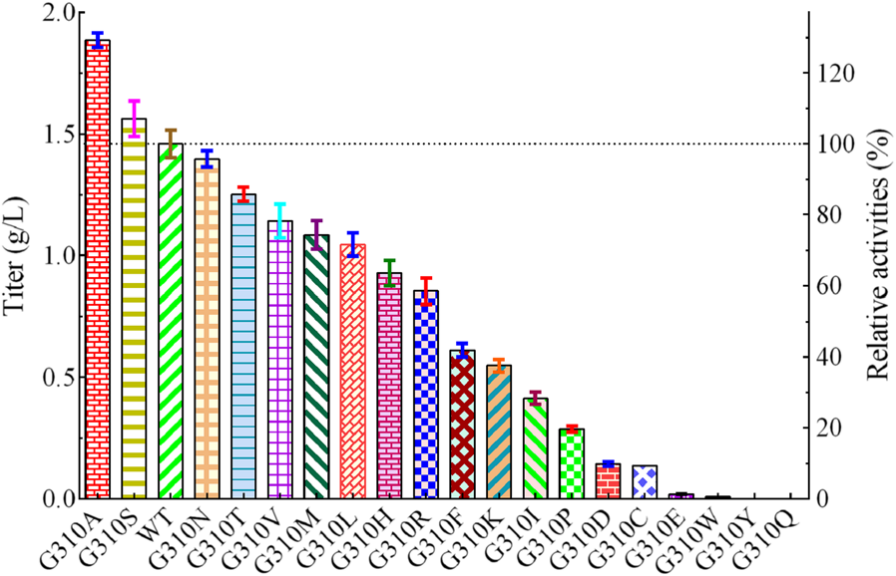
The production performance of L-carnosine by G310-site saturation mutation. Each point represents the average of three data, where the error bars represent the standard deviation. The left axis is the titer and the right axis is the relative activities. The activity data from that of wild-type (titer, 1.46 ± 0.06 g/L) was set as 100%

The above results declared that the residue site of G310 seems to be more flexible than others regarding to their influences on enzyme activity. Thus, saturation mutagenesis at the position of G310 was conducted aiming to obtain better mutations. As shown in Fig. 5, the mutant G310S can also improve the enzyme performance (titer, 1.56 ± 0.07 g/L), although it was not as effective as G310A (titer, 1.89 ± 0.03 g/L). However, the activity was decreased in other cases. Significantly, the activity was completely destroyed when glycine (G) was replaced by tyrosine (Y) or glutamine (Q).

Yin et al. [2] reported divalent metal ions strongly promoted the synthetic activity of dipeptidase. However, in this study, the divalent metal ions did not significantly stimulate the activities of gene_236976 product and the mutant G310A, although the addition of 0.1 mM Ca^2+^ increased the activities slightly (data not shown).

The enzyme kinetic parameters of wide-type and mutant were determined using steady-state kinetic methods. The saturation curves of gene_236976 product (WT) and mutant G310A for β-AlaOMe and L-histidine are fitted according to the Michaelis–Menten equation in Origin software (version 8.0), and both reached near the saturation values. The kinetic parameters were recorded in Table 3. The *K*_*m*_ of WT was 1.35 ± 0.20 mM for β-AlaOMe and 235.3 ± 76.75 mM for L-histidine, the *k*_*cat*_ of WT was 248.67 ± 7.33 s^−1^ for β-AlaOMe and 1013.33 ± 195.33 s^−1^ for L-histidine. G310A showed a much higher affinity to L-histidine (*K*_*m*_, 7.73 ± 0.29 mM) than WT, and it resulted in 4.18-fold improvement in *k*_cat_/*K*_m_ toward L-histidine (*k*_cat_/*K*_m_, 18.02) compared with WT (*k*_cat_/*K*_m_, 4.31), which clearly indicated G310A has much higher activity under this experimental condition. The results confirmed again that β-AlaOMe served as the better substrate for L-carnosine synthesis, while L-histidine gave more contribution than β-AlaOMe for the enzymatic activity since the kinetic parameters for β-AlaOMe did not change. Considering the fact that mutant G310A was the one exhibiting the best performance in the site-directed mutagenesis, it was applied in the subsequent experiments.

**Table 3:** Kinetic parameters of L-carnosine synthase (WT) and the mutant G310A

	β-alanine methyl ester hydrochloride^a^			L-histidine^b^		
	*K*_m_ (mM)	*k*_cat_ (s^−1^)	*k*_cat_/*K*_m_	*K*_m_ (mM)	*k*_cat_ (s^−1^)	*k*_cat_/*K*_m_
WT	1.35 ± 0.20	248.67 ± 7.33	184.20	235.3 ± 76.75	1013.33 ± 195.33	4.31
G310A	0.81 ± 0.04	141.33 ± 1.33	174.48	7.73 ± 0.29	139.33 ± 0.67	18.02

The kinetic parameters were measured with different substrate concentrations

^a^50 mM L-histidine with 0–1 M β-alanine methyl ester hydrochloride (β-AlaOMe)

^b^100 mM β-AlaOMe with 0–400 mM L-histidine

### Microbial production of L-carnosine by the engineered enzyme in whole-cell reaction

**Fig. 6:**
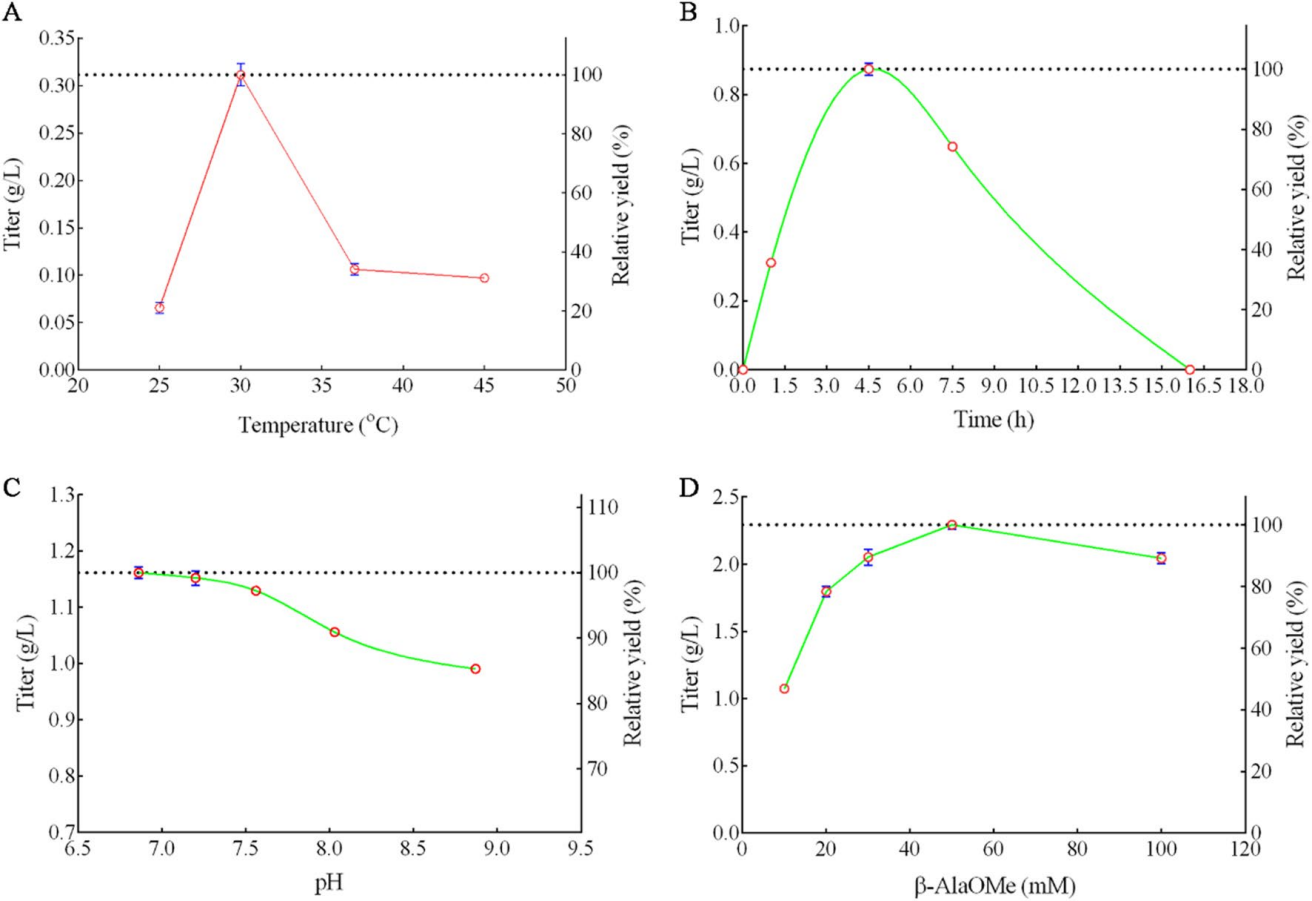
Optimization of L-carnosine production by the engineered enzyme in the whole-cell reaction. **A**, Different temperature. **B**, Different reaction time. **C**, Different initial pH. **D**, Different ratio between β-AlaOMe and L-histidine. The left Y-axis represents titer and the right Y-axis represents the relative yield. The highest titer was set as 100%. The reactions were performed by the whole-cell with a final OD_600_ = 20 in a buffer of 100 mM Na_2_CO_3_. The substrate concentrations were tentatively set at 10 mM β-AlaOMe and 50 mM L-histidine, except for the figure D, in which 10, 20, 30, 50 and 100 mM β-AlaOMe were respectively added with 50 mM L-histidine

A whole-cell biocatalyst was constructed for L-carnosine production in this study. The conditions affecting L-carnosine yield were investigated. The optimized parameters were first demonstrated by measuring the final L-carnosine production titers under different conditions by the whole cells (Fig. 6). The enzymatic reaction temperature was first evaluated by measuring the L-carnosine production titers in 1.0 h between 25 and 45 °C. The reaction performed at 30 °C by the whole-cells produced the highest level of L-carnosine (titer, 0.31 ± 0.01 g/L) (Fig. 6a). And the optimized reaction time was found to be 4.5 h under 30 °C (titer, 0.87 ± 0.02 g/L) (Fig. 6b). The final production titers obviously decreased after 4.5 h, indicating that the enzyme also has hydrolysis activity towards L-carnosine. Then the hydrolysis activity on L-carnosine by the purified enzyme was confirmed (data not shown). Heyland et al. [17] reported the L-carnosine formation was most effective at pH 10.0 by the enzyme of DmpA in *E. coli* whole cells, while 8.0 was chosen as the most suitable reaction pH for *Sm*PepD, the dipeptidase from *Serratia marcescens* in the purified enzyme-catalyzed reaction [2].

Thus, we first tried to test the reaction at pH 10.0 in a buffer of 100 mM Na_2_CO_3_. However, the initial pH values decreased a lot when the substrates of β-AlaOMe and L-histidine were added. To accurately describe the actual value, we re-measured the initial pH at the beginning of the reaction, irrespective of the calculated values from the standard buffer solutions. As shown in Fig. 6c, the highest titer was obtained in the initial pH 6.86 (titer, 1.16 ± 0.01 g/L) for the enzyme characterized in this study, indicating the different reaction parameters from different enzymes. To promote the reaction equilibrium in the direction of the L-carnosine synthesis, the ratio between β-AlaOMe and L-histidine was also investigated. Since L-histidine is a more expensive substrate and was already added at a high concentration of 50 mM in the reaction system, only the concentrations of β-AlaOMe were varied to check the effects on the L-carnosine production. Since the β-AlaOMe concentration increased in the reaction, we also extended the reaction time and sampled both at 4.5 h and 6.0 h, respectively. The titers at 6.0 h were all higher than those at 4.5 h. Then we just showed the reaction time data of 6.0 h in the text. With the increase of β-AlaOMe from 10 to 50 mM, the L-carnosine titer increased accordingly and reached the highest of 2.29 g/L (10.12 mM) at 50 mM β-AlaOMe added, with a molar yield of 20.24% (Fig. 6d).

## Discussion

Carnosine is known to be synthesized from β-alanine and L-histidine by an ATP-dependent synthase (EC 6.3.2.11), which has been partially purified from different sources. Drozak et al. [6] identified a “ATP-grasp family” ligase, which served as L-carnosine synthase in chicken muscle. Thus, we first concentrated on this enzyme. The gene was codon-optimized, synthesized, and expressed in *E. coli* and yeast, respectively. However, although we have tried lots of efforts and finally achieved the expression of soluble enzyme, no activity towards carnosine synthesis from β-alanine and L-histidine with ATP was confirmed (data not shown). The same results were obtained with the orthologous enzymes from mice and oysters, as indicated in the literature [6]. Given the fact that the enzymes all come from eukaryotic cells, which should not be easy to get active in microbes, we shifted the direction to microbial origins from the viewpoint of industrial application interests. Using the enzyme DmpA from *O. anthropic* expressed in *E. coli*, a simple enzymatic procedure for L-carnosine synthesis was developed by whole-cell biocatalysis, although the enzyme uses β-alanine-amide, but not β-alanine, as one of the starting substrates [17]. This result gives an indication that more enzymes may exist in nature for the efficient synthesis of L-carnosine, which is also suitable for the microbial synthesis process. Thus, the deep-sea sediment metagenome was used in this study, and a novel L-carnosine synthase gene, gene_236976, was successfully mined. It shared only 14.3% identity in amino acid level with currently reported dipeptidases (*Sm*PepD) from *S. marcescens* by genome mining approach [2], which showed relatively high synthetic activity toward L-carnosine (space–time yield of 60.3 g/L/d).

Although gene_236976 product showed the activity for L-carnosine synthesis using β-alanine-amide and L-histidine as substrates, β-alanine-amide is a relatively much expensive substrate compared to β-alanine. Thus, exploring cheap and easily accessible feedstock is a promising way to increase the process economy. According to the results, β-alanine ester could be the more suitable substrate for L-carnosine synthesis and showed a promising cheap feedstock for an industrial setting. Except for the exploration of inexpensive substrates, increasing the enzyme activity of gene_236976 product is an effective alternative way to increase the yield of L-carnosine. Therefore, mutagenesis was performed according to homology modeling and molecular docking. Finally, a mutant G310A performed best and was applied in the subsequent experiments.

**Fig. 7:**
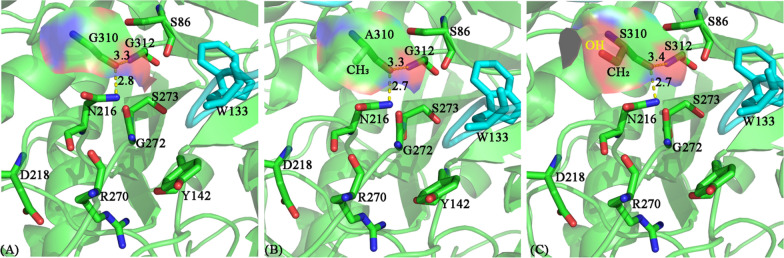
Comparison of the partial potential active site of L-aminopeptidase and its mutants based on L-aminopeptidase from *O. anthropic* (PDB code: 1B65). A-C: Stereoview of the potential active site of L-carnosine synthase (WT), G310A, and G310S. The asymmetric unit is colored in green and cyan, separately. The positively and negatively charged regions of molecular surface at residue 310 are colored in blue and red

When analyzing the conformation of all amino acids at predicted active site, it is clear that only the mutant with minimal structural change (no change of stereochemistry or bulky side-chain), such as mutants G310A and G310S, could result in increased enzymatic activity (Fig. 7). In particular, mutant G310A bearing the additional CH_2_-group exhibited the highest relative activities (titer, 1.89 ± 0.03 g/L), indicating that the additional CH_3_-group might play an important role in increasing enzymatic activity through the potential hydrophobic interaction (Fig. 7B). Whereas, the extra -OH group of mutant G310S led to a distinctive increase in the negatively charged region (red region, Fig. 7C), which might produce some negative effect on enzymatic activity. Thiyagarajan et al. [31] reported that the additional methyl group at the catalytic subsite would result in steric hindrance for substrate binding. Here, our studies, including the kinetic parameter data, further highlighted that the additional CH_3_ group of mutant G310A or the potential hydrophobic effect caused by such group was the main factor influencing the substrate-binding affinity and enzymatic activity.

Biocatalysis can be either performed by purified enzymes or naturally encapsulated in whole cells. The use of whole cells in biotransformation can be relatively simple but also has limitations, including the reduced enzyme activities or unspecific side reactions compared with the free-enzyme reactions. Thus, the whole-cell biocatalytic process should be thoroughly evaluated in order to develop an economically attractive solution [17]. The aminopeptidases catalyze a reversible reaction and the pH, temperature, reaction time, and concentration ratio of substrates are all factors that could affect the equilibrium [8, 9]. In this study, the temperature and pH were optimized, and the yield of L-carnosine was increased significantly.

In conclusion, a metagenome mining approach from deep-sea sediment was successfully identified a new aminopeptidase (gene_236976) with synthetic activity toward L-carnosine. β-Alanine methyl ester was proven to be the best substrate for the synthesis, and the enzyme activity was increased by structure-guided rational design. This study enriched the enzyme information for developing the microbial synthesis process of L-carnosine production.

## Supplementary Information

**Additional file 1**: **Note S1** The amino acid sequences of 22 homologs

**Additional file 2**: **Fig S1** Phylogenetic tree analysis of the 22 homologs

**Additional file 3**: **Fig S2** Amino acid sequence alignment of the 22 homologs mined from deep-sea metagenomic data

**Additional file 4**: **Fig S3** SDS-PAGE profile of purified L-aminopeptidase by Ni–NTA agarose.

## Data Availability

The data supporting the findings of this study are available within the article and its supplementary information files.
