# Supplementary material for: Characterization of a new L-carnosine synthase mined from deep-sea sediment metagenome
Source: Microb Cell Fact. 2022 Jun 27;21:129. doi: 10.1186/s12934-022-01854-w (PMC9235088; doi:10.1186/s12934-022-01854-w)
Supplement: Supplementary file 1 — Additional file 1: Note S1 The amino acid sequences of 22 homologs [file 12934_2022_1854_MOESM1_ESM.docx]

Supplementary File

**Characterization of a new L-Carnosine Synthase Mined from Deep-sea Sediment Metagenome**

Jiajia She^1,2^, Lihong Fu^1^, Xiaowei Zheng^2^, Jing Li^1,2^, Limin Wang^2^, Bo Yu^2^, Jiansong Ju^1*^

^1^ College of Life Science, Hebei Normal University, Shijiazhuang 050024, China

^2^ Institute of Microbiology, Chinese Academy of Sciences, Beijing 100101, China

*Corresponding authors.

E-mail: [jujiansong@126.com](mailto:jujiansong@126.com); (J. Ju)

**Supplementary Notes**

**Supplementary Note 1 The amino acid sequences of 22 homologs**

The enzyme of gene_236976, which shared an identity of 44.8% with reported aminopeptidase (DmpA) from *Ochrobactrum anthropic*, was shown in the first one.

>gene_236976-identity 44.8%

LIISSVSAAEPIRARDLGIPFDGQPGSLNAITDVAGVEVGQVTLIDGEGALLVGSGPVRTGVTVIHPRGRNSTDPVFAGWFALNASGEMTGTTWLEERGMVDGPIAITNTHSVGVVRDAAVAWMVEQGWPADWHAPVVAETYDGGLNDINGFHVTREHALEAMAKARTGVVEEGVVGGGTGMVCNGFKGGIGTSSRVFDALGRSFTVGILVQCNYNWDGEQDLRIGGKNMSGLLPVGKHCFIYRDVPRHVNWYPYCDDSSANDELDKPTRDGSIIIIVATDAPLLPHQLRRLAKRPALGLGRLGGISSDGSGDIFLAFSTASPGLINENEESTISMFPNNGLSVVFEAAVQATEEAIVNAMVAAETVVGASGLQVEEMPEDQLRAIFLD

>gene_1241-identity 50.5%

LLPIAVIADDALRARDLGIPFDGTPGPLNAITDVPGVTVGHSTVIEDHNNSSAARTGVTAILPRGEDSVMLPVFGGTAVLNGAGEMTGTIWVEASGFLEGPVMITSTQSVGMVFEETIKWRVNHAERDVTGYAWSDPVVAETWGGRLNDENAFYVRPEHVIEAIETATSGPVAEGNVGGGTGMACYQFKCGIGTASRVVETVDGKFSVGVLVQANYGWRDILQIAGVPVGREMPVVQVADIRTPMDDEMGSIIVVIATDAALLPHQLKRVATRAGLGEARVGGMASNGSGDIFIAFSTANPQELRAGTNLSVRLLGNEHVTPVFSGTVLATEEAIVNALVAARTMTGFEGRTIEAIDHEALRAALRKYNRLDEPQ

>gene_68362-identity 51.6%

MIALDPASSTSRRALQCAAASLVIFLCFAVSATAQKPRARDLGVPFEGVPGALNAITDVEGVEVGHATLIRGSGPKIVGQGPVRTGVTAVWPRGNDTPDPVFAAWFSLNGDGEMTGTTWVQDSGLLEGPVMITNTLNVGIVHHAVVRWGATRSVDYEGGGYPPWLVSLPVVAETWDGTLNDILGQHVTVEDALAAMDNASGGRVEEGSVGGGTGMICNRFKGGIGTASRVVQIGGEGYTVGVLVQCNYGDRAPLRIAGVPVGQEIPDLLPSRPGANGDPVRADDGADRAGSIIVVAATDAPIMPHQLERVARRISLGLARNGSTSGNGSGDIFIAFSTANREAARERAVTSGVEILANGRLNPIFAATVEATEEAIVNAMVAAETMTGADDVTVYALPHDRLREALRKYNRLGG

>gene_241831-identity 50.4%

LGIPLLGTPGSFNAITDVLGVEVGHATLVEGQGKLVVGKGPVRTGVTAILPRGKTYDPAFAGWYSLNGNGEMTGTAWVEESGFLESPVLITNTHSVGVVRDAVIAWRYEHDCCESLPDRQGVFWSLPVVAETYDGILNDINGFHVKPEHVFAALDGAASGAVREGNAGGGTGMICHQFKGGIGTASRLIEIESKTYALGVLVQANYGARSDLTIAGVPVGRAITDLTPELQSGNPDIKSGSIIVVVATDAPLLPHQLKRLARRVPLGIAKMGGNGENTSGDIFIAFSSANPESSKRVGLKQLTMLPNDQMDPLFQATVQGTEEAIINAMLAAETMTGIDGNTVHALPHDRLQVALKKYNRLL

>gene_281166-identity 48.9%

MTDPHRATPSGKARARALGAPFDGAPGENNAITDVPGVAVGYSTIIRGDGPLVVGRGPVRTGVTAILPRGHGNFTIPVFAGAFSLNGAGEMTGTIWIEESGQCEGPITITNTHSCGMARDATIRWIVDHYPAELADAWGLPVAGETYDGDLNDINGFHVAPEHVYEAIDSADGGAIEMGSVGGGTGMICYDFKGGSGSASRLVGEYTVGAFVQANFGVRSELVIAGVPVGKHITGGEVRGKPGGSIIAVVATDAPLMPHQLKRLARRVGLGVGRSGSIAHHGSGDIFLAFSTANRAAWARGSGPRDCRFLPNDAMDPLFAGVVEATDEAIVDSMVANETMTGRDGVTAIALPHDRLSEILAEHNRLSKSS

>gene_366662-identity 44.6%

MPTGEHNAITDVPGVLVGHTTVVHDEPRVARTGVTMIIPREGAIANDRAFAGYHSLNGNGEMTGLLWIEESGILDSPIGITNTHQVGVVRDALVAHAIDKGHPGWVLPVVAETYDGFLNDIGAFHVTQDHVFQAIASARSGPVEEGNVGGGTGMTCHEFKGGIGTSSRIAETETGQYVVGALVQANYGTRRLLRVDGVPVGREIGSEHTPTPWQSSSGEGSIIVIVATNAPLIPAQCKRLAQRATVGLARVGGVGHNGSGDIFLAFATGNHLPFNSSALHDLKMLPHEQINSIFEATAEAVEESILNALTAAETTTGFQGHTAHALPLDELKRVMAKYRPST

>gene_454500-identity 49.6%

LDFSYYSFERKPKMKKLIFTSSLLLVCLSTYAQKPRARDLGIPFEGTPGPLNAIIDVKGLEVGHSTIISGSGKNVLGKGPVRTGVTAIFPRGKQNKFSPVYANWYSLNGNGEMTGTTWVTESGFLETPIMITNTNSVGVVRDAVLKWFVDTNWYKDENWWYTYPVVGETYDGFLNDIYGFHVKEEHVLEAIENASSDNVQEGNVGGGTGMMCLGFKGGIGTASRIVSIQDQDYIVGVLVQSNFGARRNLTIAGVPVGKELIDTLKTEFKAPPQSRRQESDGSIIVVVATDAPLLPHQLKRIAQRVPLGIGIVGGRGSNGSGDIFIAFSTANKNAFDRSDNQSVVTLSNDRITPLFEATVQSVEEAIINAMVAAETMEGNNGNKAYALPHGALVEILRKYNRLK

>gene_454672-identity 51.6%

MTTHMPSLKRLRSHRAGWLWLTLLLTAQSAFAQDKPRARDLGIPFEGTPGPSNAITDVAGVFVGHTTLISGEGPLTIGEGPVRTGVTAILPRGDDPADPVFAGWFALNGNGEMTGTTWVEESGFLEGPIAITNTHSVGVVRDAIIAWQIERGAAFQPWSLPVVAETYDGFLNDINGFHVKAEHLFSALDNARSGPVAEGNVGGGTAMTCLGFKCGIGTSSRTGLGPGGYTVGALVQANFGGRRQLTVAGVPVGREIVREQRARPAPESAGRGSIIIIVATDAPLLPHQLKRIARRASLGVARTGGTAGNGSGDIFVAFSTANAESAGGRPTSDIQMLSNSRITPLFDATVQAVEEAIINALVAAETMVGRDGNRAEALSHERLREILGRFNRLAG

>gene_464923-identity 48.3%

MLAQKIVALTVAAQVISTPAALVFIASLLAVAAPAMAQQRARARDLGVPFDGSPGPLNAITDVGGVRVAHTTLIQGEGELRVGEGPVRTGVTVIFPRGDDPADPVFSGWFALNGNGEMTGTTWIEESGFLEGPIAITNTHSVGVVRDAIIGWQAERGSGFQPWSLPVVAETYDGSLNDINVVHVRTEHVYSVLENAASGPVDEGNVGGGTGMRCLGFKCGIGTSSRIVEVGGETYTVGVLVQANFGGRRQLTVAGVPVGREIMREDEAQRDEAPAPEGPEAAAETPESAHTREAVEAREAEDIETDRGSIIIVVATDAPLLSHQLKRIARRASLGVARTGGTAGNGSGDIFVAFSTANPGAASARPVAELTMLSNSRISPVFDATVEATEEAIINALIAAETMVGRDGNRSEALDHDRLREILARYNRLAQ

>gene_520036-identity 49.5%

LAEDSPRARDIGIPFDGTPGKWNAITDVTGVTVGHHTMMEDLPDGKAVRTGVTAVLPLGRQSLMRPVFAGWFSLNGCGEMTGTTWLEESGQLEGPVMLTNTHSVGMVHHATIAWRVRQGGPDASGYFWSAPVVAETWDGYLNDINGFHVDPNHVDAALEGAASSPVTEGNVGGGTGMVCHGFKGGIGTSSRVIEILNESYTVGVLVQANYGARRDLRIAGVPVGKHLQKKRASSQSKPDAEGDGSIIIVIATDAPLLPYQLKRLAKRAGMGLARMGSVANNGSGDIFIAFTTADQSLGRVDRLLGHRSVPNDRMNPLFAATVQATEEAIVNALVAARDMTGDRGHHITAIPHDELVEVLSRYRMIIER

>gene_619370-identity 50.8%

MKNALLTLLLLPLLAVADPRARDLGIPFEGTPGEFNAITDIAGVTVGFESIIRDLPNGKAVRTGVTAILPRGAASNDQPVFGGWFSLNGNGEMTGTTWVEESGFIEGPVMITNTHSVGAVHEGVIKWRYEHGAADASGFWWSLPVVAETWDGYLNDINGFHVRPEHAFAALNSAKSGPVAEGNVGGGTAMVCFEYKCGTGTSSRVTQAAGDDYTVGVLVQANFGSRDYLLVAGVPVGKLMRDNMVYSNDEPAVEETGSIIIIVGTDAPLMPHQLKRLTRRAALGLSRTGSYAGNGSGDIFIAFSTANENANQSNSDVSLDTLSNEDMNPLFIATIQAVEEAITNALIAGRDMTGNRGNTVSAIDHEQLKSLLRQYNRLSE

>gene_673701-identity 49.9%

LDGTPGDLNAITDVSGVEVGHTTLIQGEGELRVGEGPVRTGVTIVLPRGKDSSDPVFAGWFSLNGNGELTGTTWVEESGYLEGPIAITNTHSVGTVRDALTAWSIRHDKLLQHWSLPVVGETWDGDLNDINGFHVKAEHVYDALDSATAGSVAEGNVGGGTGMICYEFKGGIGTASRRLSKDEGAYTVGVLVQSNFGLRNQLTIAGVPVGREIPEIASASRDERGSIIIVVATDAPLLPHQLKRLARRASLGVARTGGVSGNGSGDIFIAFSNANPGTAGAEQRAQLEALSNSRMDPLFKGTVEATEEAILNALVAAETMTGINDHTVTALPHRKTREVLKQYGRLSD

>gene_738131-identity 49.7%

MIRRTVFTVFTIFTVFTIPSSIEAQSKPRARDLGVPFEGSPGTLNAITDIAGITVGHVTIIRGEGRLVVGEGPVRTGVTAILPRGMSNDPVFAGWYSLNGNGEMTGTTWVEESGFLEGPVMITNTHSVGIVHDAVIEWNRDHRRQFQLAPTSWWSLPVVAETWDGGLNDINGFHVKKEHTFEALDGAQAGPVAEGSVGGGTGMRCHQWKAGIGTSSRVVGDYTIGVLVQCNYGSRRSFRVAGVPVGMEIPDMLPQAGNLGGDNDDLGSIIVVVATDAPLLPHQLKRLARRVPMGIGRVGGYAGNGSGDIFIAFSTANEGAADRREIQTLEMYPNDRMSGLFEATAQAVEESIINAIIAGETMVGINGNTTYGIPHGRLQEVLERYNRLER

>gene_784591-identity 51.8%

MGVADDAVPRARDLGIPFDGTPGPLNAITDVPGVTVGHETIVRDLPDNKAVRTGVTAILPRGVASNNQPVFGAWFTLNGNGEMTGTTWLEESGFLEGPVMITNTHSVGAVHQATIQWRIDQGAADSSGYWWSLPVVAETWDGYLNDINGFHVKGEHARAALDAAKGGSVPEGSVGGGTGMICHGFKCGIGTSSRIAETNEGSYTVGVLVQANYGSRDLLRIAGVPVGRHLQEDRPFAKLQNPQFAAGSIIIVVATDAPLLPHQLKRLLRRAALGLARNGSIASNGSGDIFIAFSTANKDADQAGAGVTVRTLANENINPMFLATVEATEEAIINALIAGRDMRGHRGNIVKAIEHDDLRSVLRDYNRLEETK

>gene_1065070-identity 57.9%

MAKPRGRDLGLPFPGETGPNNAITDVPGVMVGATTRIEGEGPLVPGQGPVRSGVTAILPLGKGGEPQPVWAGIYALNGNGEMTGSHWVTDGGYFVGPICITNTNSVGIVHHAANKWIIENHREDWEDAHLWAMPVVAETYDGVLNDINGQHLTEADALAALDSAKPGPVSEGNSGGGTGMVCYEFKGGTGTASRQIDVEGKRYVMGALVQANHGTRPWFAPLGVPVGRHMTENRISLGREQGSIIVVLGTDAPMLPHQLQRVARRAAIGVGRGGSPGGNNSGDIFLAFSVANRMPMMQMAPHHLKLDFLNDEIFDDIYLAAVEAVEEAVLNAMVAAEDMTTLRPAGKICRALDHQQLVEIMRRYGRCS

>gene_1141084-identity 50.9%

LRNLLLVFCLLPFAASAAESPRARDLGVPFDGTPGPLNAITDVPGVTVGHATIIQDYENGSAARTGVTAVLPRGMDSVMLPVFGGTAVLNGAGEMTGRIWVEASGFLEGPVMITTTQSVGMVFEETIKWRVKHAEPDVTGYAWSDPVVAETWGGQLNDENGFYVRPQHVIEAIESAQPGPVAEGNVGGGTAMTCYQFKCGIGTASRVVKTVDGDFTVGVLVQANYGWRDILQIAGVPVGREIPVEQVADVRTPMDDEKGSIIVVIATDAPLLPHQLKRVATRAGLGIARVGGMASNGSGDIFIAFSTANPRELRSGSNLSVSLLGNEHITPVFSGAVLATEEAIVNALVAARTMTGFEGTTVEAIDHAALRAALRKYNRLDESQ

>gene_1221824-identity 46.9%

MKKIINTFLISLAFYSAALGQQSRARDLGIPLDGIPGELNAITDLKGVEVGHTTLISGDGELIVGEGPIRTGVTAILPRGKKYDPVFAGWYSLNGNGEMTGTTWVEESGFLEGPIMITNTHSVGIVRDAVIEWQYKNKYFDPLPNQPDVFWALPVVAETYDGTLNDINGFHVKKEHVFSALDEAKSGKVEEGNVGGGTGMICHRFKGGIGTSSRILTINGNKYTLGVLVQANYGNRESLTISGVPVGKEIADLMPENDPLKKDLDQGSIIVVVATDAPLLPNQLKRLARRVPIGISRMGGFASNGSGDIFIAFSTANAGAANRKENQNILMIPNDKMSALFEATAQATEESILNALISAETMIGKNNSKVYELPEDRLIEVLKKYNRIK

>gene_1377453-identity 50.9%

MRRSVSILAVLGIHLALLGAATVQGQTKPRARDIGVPFEGSPGPLNAITDVAGVAVGHVTLIEGEGPLQVGVGPIRTGVTAIRPRAQTNLPVFAGWYSLNGNGEMTGTTWVEESGFLEGPVLITNTHSVGIVRDAVIEWMRDEEHIRPVIPGVWWGLPVVAETYDGSLNDINGFHVRKEHAFQALNTAAEGPVAEGAVGGGTGMICNRFKGGIGTASRRVDGYTVGVLVQCNYGGRDGLTIAGVPVGREIRDLLPELRYDARGENGSIIVVVATDAPLLPHQLKRLARRVPIGIGRVGGVGTNGSGDIFIAFSTANPDAWSRKETVDLEMLPNDAMSSLFKATAEATEESIVNAMVAAETMVGINGNKVYALPHDRLQETLREYNRLELPQ

>gene_1422059-identity 50.4%

PGIQLGRNQMSVTACIRLAFCQIKVLLIVLTCAVPILAFADDKPRARDIGIPFAGTPGPYNAITDVAGVTVGHTTLISGDGKLDVGSGPIRTGVTAIHPRGKTYDPVFSGWYALNGNGEMTGTTWVEESGFLEGPVVITNTHSVGVARDATIEWQYTNNLFDPLPDDPDVFWSLPVVAETYDGDLNDINGFHVTKEHVIDALESAKPGRVAEGNVGGGTGMICHQFKGGIGTASRRLKVEGSEYTVGILVQANYGIRETFTIAGVPLGDKLSDLMPIFRPTDRGTGSIIVVVATDAPLLPHQLERLARRVPLGIAKVGGYASNGSGDIFIAFSTANPGAATREGLKDIEMLPNDSMSRLFLATAQATEEAIVNALVAAETMVGINGNTVYALPQDRLVSILQKYNRIP

>gene_1625963-identity 46.7%

MPQRCRPIATLVLLALTVAIAADAQTRPRARELGVPFEGSPGTLNAITDVANLTVGHVTIIEGEGRLVVGEGPIRTGVTAILPRGMTNDPVFAGWYALNGNGEMTGTTWVEESGFLEGPVMITNTHSVGIVHAAVIEWNRDHRRQYQLAPTSWWSLPVVAETWDGGLNDINGFHVTKQHTFDALNSASGGRVAEGSVGGGTGMRCHQWKAGIGTSSRLVGDYVIGVLVQCNYGSRHPFRVAGVPVGTEIPDLLPEAGNLGGDSDADVDDIRELGSIIVVVATDAPLLPQQLKRIARRVPMGIGRVGGYASNGSGDIFIAFSTANVGAADRRENQSIERLPNDRMSGLFEATAQAVEEAIINAIVAGETMVGINGNTTYGIPHDRLREVLEKYNRLER

>gene_1929613-identity 51.6%

MGLAGLAAPSTSAGQDVRARDLGIPFEGLPGPLNAITDVPGIEVGHVTIIRGDGPLVKGDGPVRTGVTAILPRGRGFDPVFAATYALNGNGEMTGIHWIEESGFLETPIMITNTFSVGVVRDAVIAWMDSTGHNAAPDGLWYTYPVVAETYDVLNDILGQHVTRAHAFEALNNASGGPVAEGNVGGGTGMIAHQFKGGIGTASRLLPSGHTLGVLVQANYGGRSRFSVAGVPVGREIPDLLPNWDVLTRGSIIVVVATDAPLLPQQLQRLAERVPLAIGRLGGLGGNGSGDIFIAFSTGNPGAWRERPAASLDMLP

>gene_1932634-identity 53.4%

MSEAHRMIAERSMATGFVINLSVLLLFSLYASAAVADPVRARDLGIPFDGTPGALNAITDVEGVTVGHETIIKDLPNDRAVRTGVTAILPRGRDSHDNPVFAGWFTLNGNGEMTGTTWVEESGFLEGPVMITNTHSVGAVHQATIAWRVAQGGPDASGYFWSLPVVAETWDGYLNDVNGFHVKPGHAQAALESAVGGPVAEGSVGGGTGMNCHEFKCGIGTSSRIARTADGEYTVGILVQANYGRRDTLRVAGVPVGQHLRNDRVFSADDEMPAEAGSIIIVAATDAPLLPHQLKRIARRTSLGLARVGGIGGNGSGDIFIAFSTANKNAGQVARGVSVRMLANEDM

**Supplementary Fig. S1 Phylogenetic tree analysis of the above 22 homologs**

The reference sequence of DmpA from *Ochrobactrum anthropic* is underlined and the enzyme studied in this text is boxed.


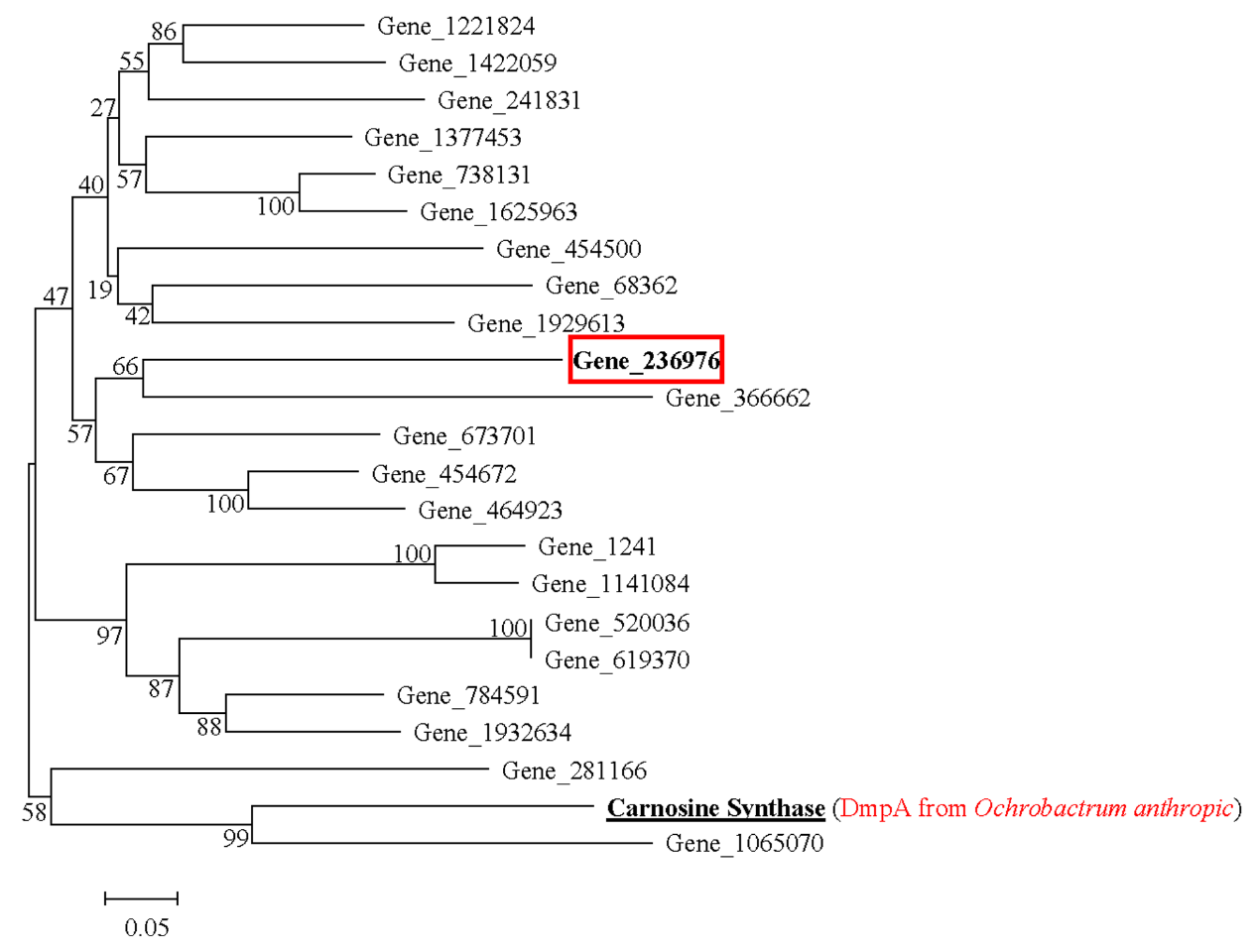


**Supplementary Fig. S2 Amino acid sequence alignment of the 22 homologs mined from deep-sea metagenomic data**

Carnosine_synthetase: DmpA from *Ochrobactrum anthropic*

**Supplementary Fig. S3** **SDS-PAGE profile of purified L-aminopeptidase by Ni-NTA agarose.**

L, cell lysates; P, precipitates; F, flow-through; W, washing; M, protein marker; E1 and E2, Elution 1&2.

**
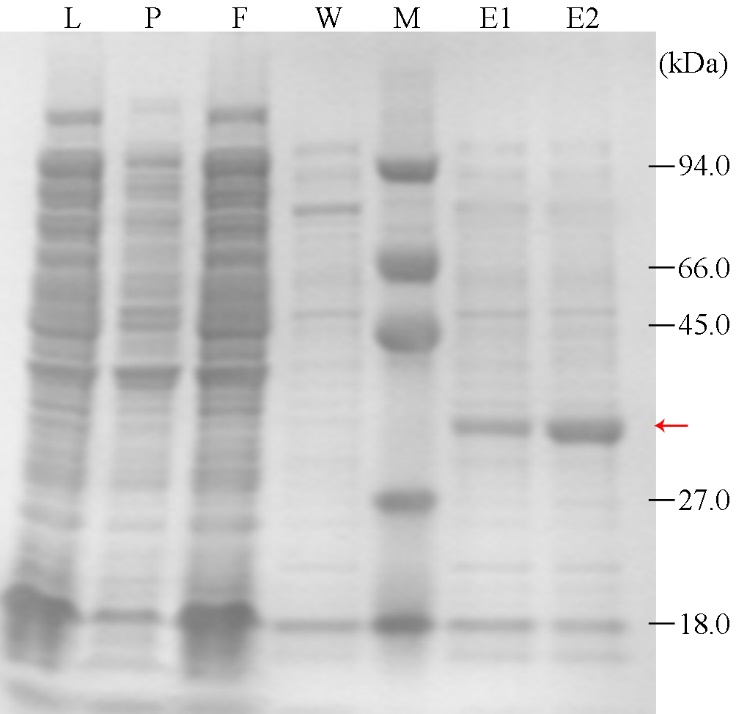
**
