# Supplementary figures and images for: Characterization of a new L-carnosine synthase mined from deep-sea sediment metagenome
Source: Microb Cell Fact. 2022 Jun 27;21:129. doi: 10.1186/s12934-022-01854-w (PMC9235088; doi:10.1186/s12934-022-01854-w)

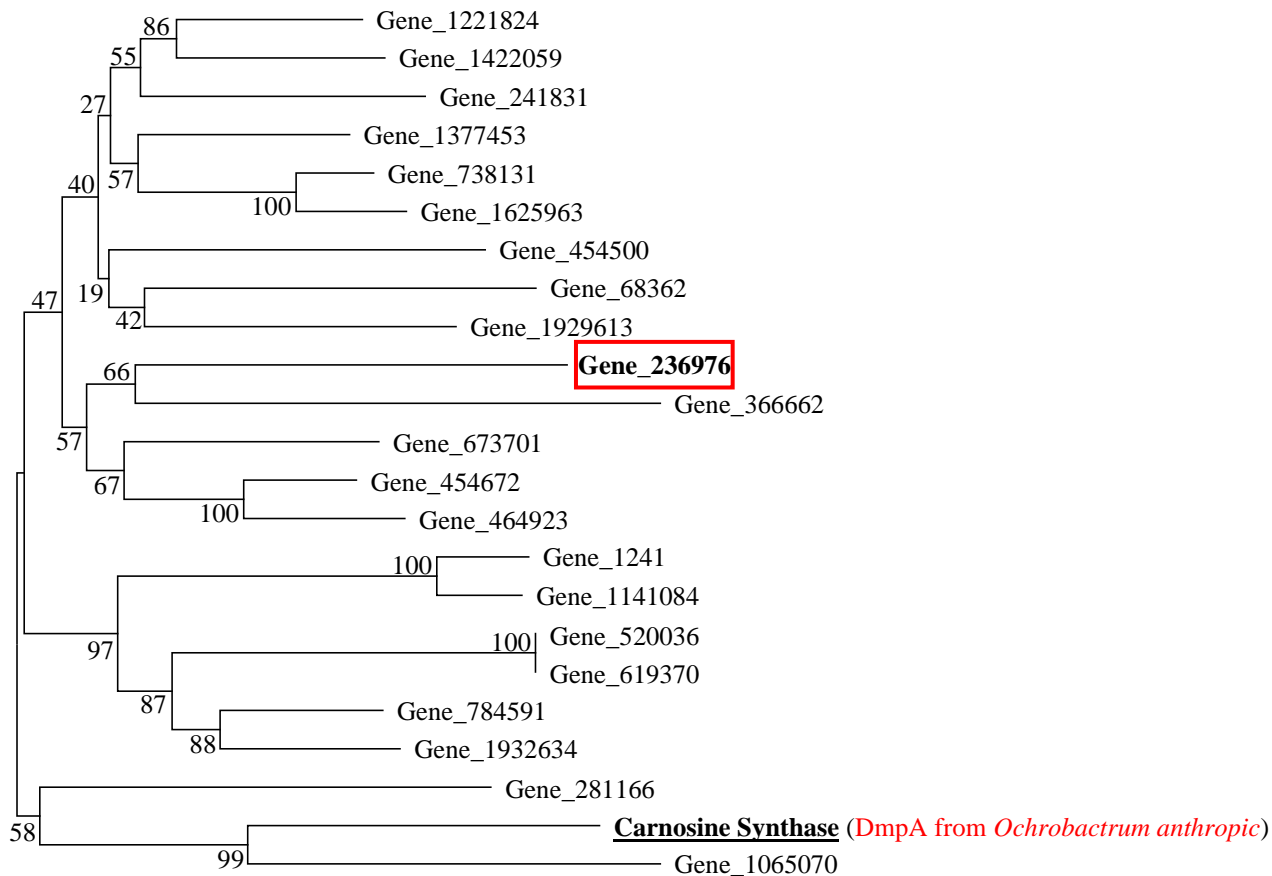

0.05

Supplement: Supplementary file 2 — Additional file 2: Fig S1 Phylogenetic tree analysis of the 22 homologs [file 12934_2022_1854_MOESM2_ESM.pdf]

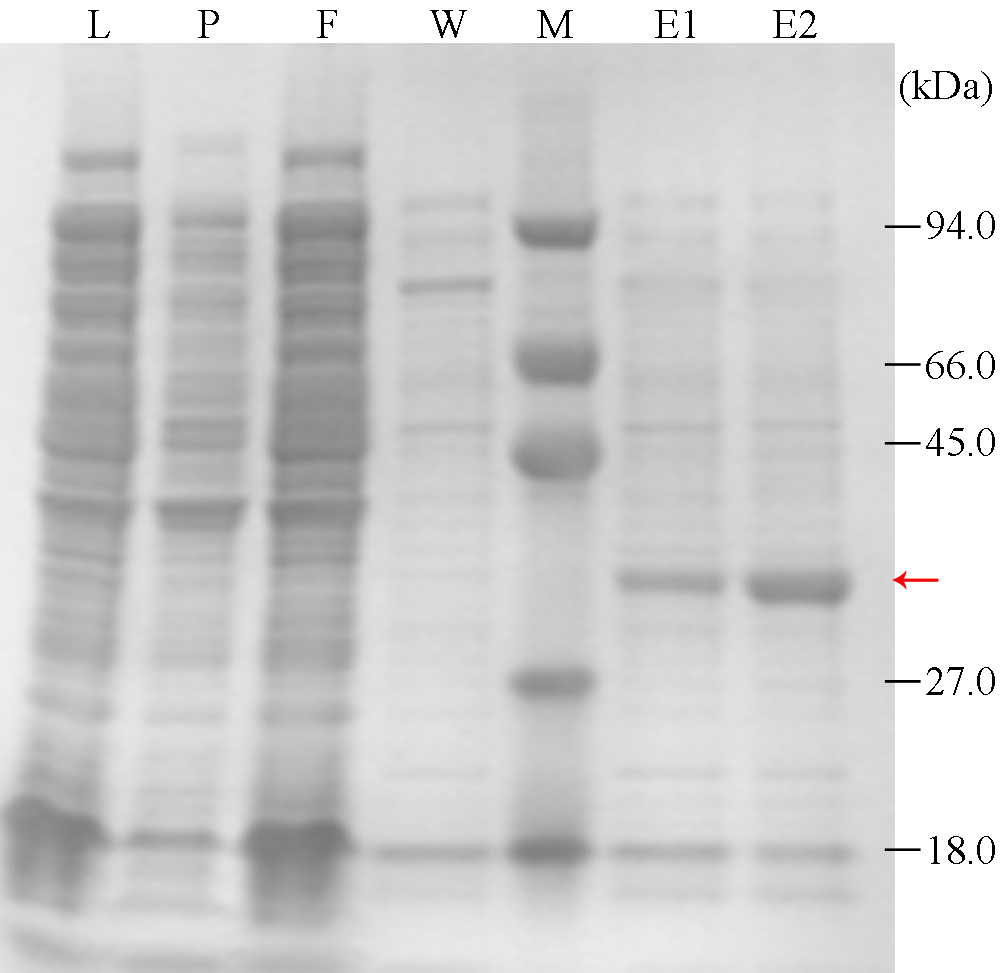

Supplement: Supplementary file 4 — Additional file 4: Fig S3 SDS-PAGE profile of purified L-aminopeptidase by Ni–NTA agarose. [file 12934_2022_1854_MOESM4_ESM.tif]
