# Supplementary material for: Characterization of a new L-carnosine synthase mined from deep-sea sediment metagenome
Source: Microb Cell Fact. 2022 Jun 27;21:129. doi: 10.1186/s12934-022-01854-w (PMC9235088; doi:10.1186/s12934-022-01854-w)
Supplement: Supplementary file 3 — Additional file 3: Fig S2 Amino acid sequence alignment of the 22 homologs mined from deep-sea metagenomic data [file 12934_2022_1854_MOESM3_ESM.pdf]

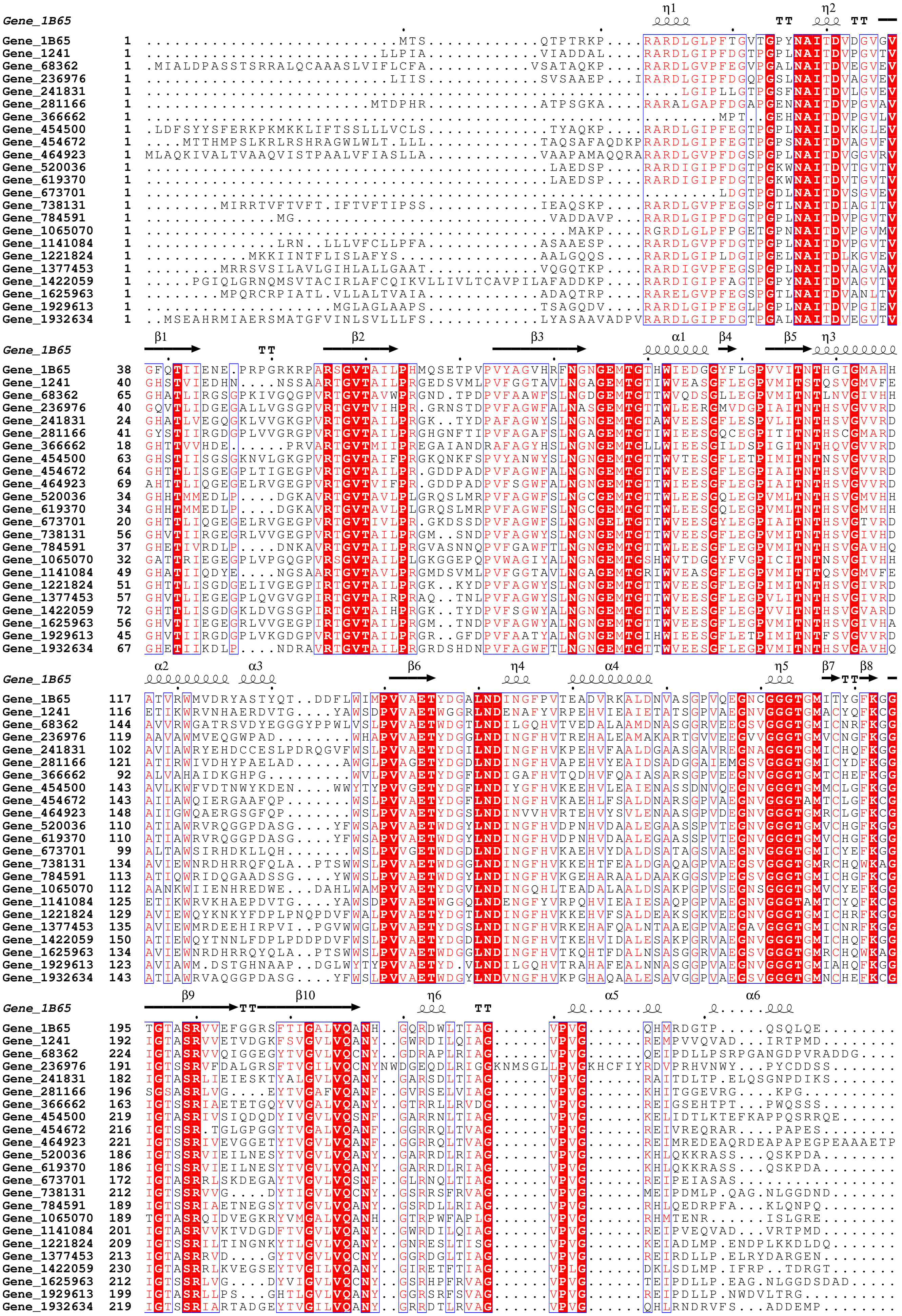

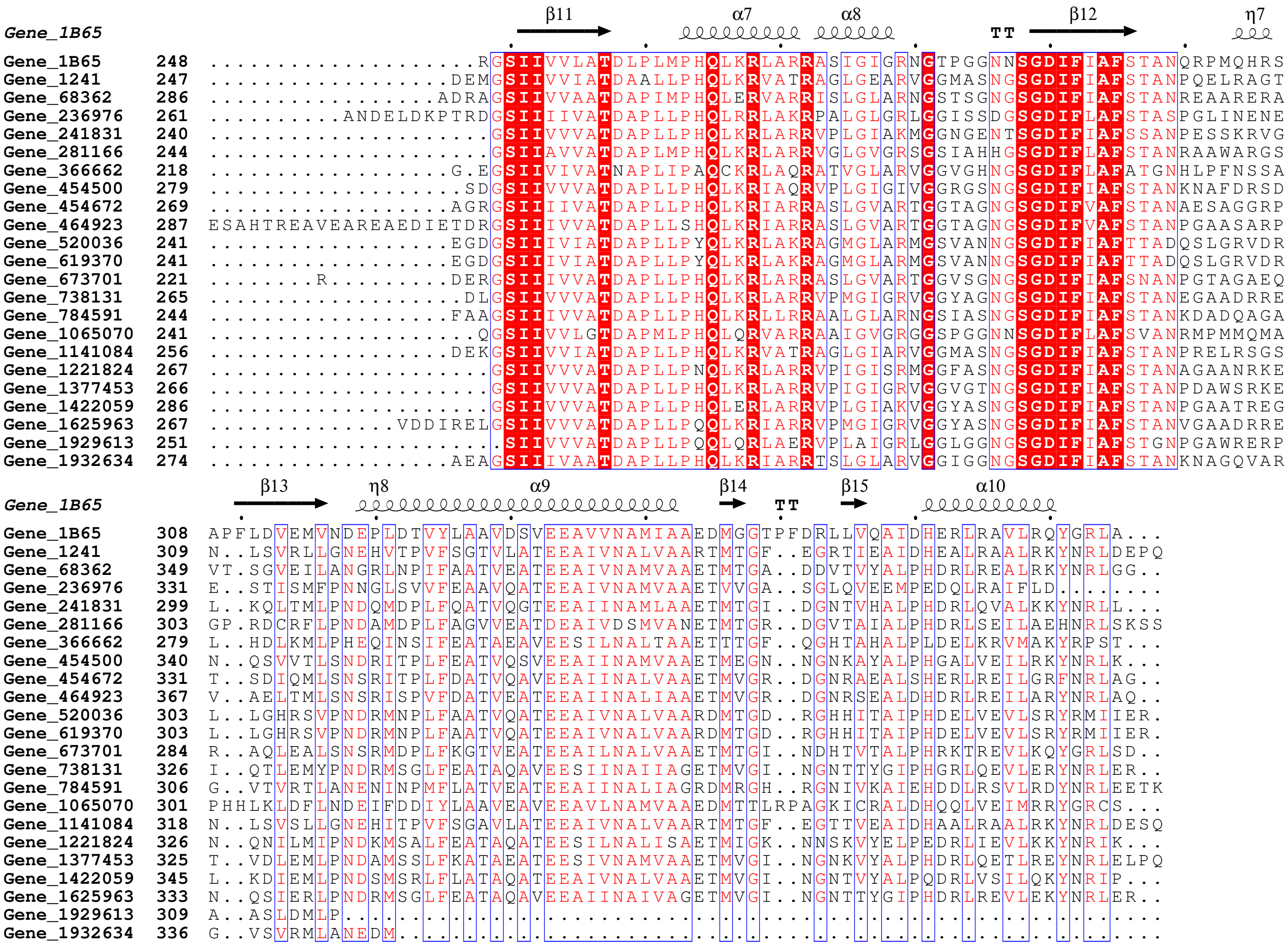

Gene_1B65
β11 α7 α8 TT β12 η7
Gene_1B65 248 ....................RGSIIVVLATDLPLMPHQLKRLARRASIGIGRNGTPGGNNSGDIFIAFSTANQRPMQHRS
Gene_1241 247 ..................DEMGSIIVVIATDAALLPHQLKRVATRAGLGEARVGGMASNGSGDIFIAFSTANPQELRAGT
Gene_68362 286 .................ADRAGSIIVVAATDAPIMPHQLERVARRISLGLARNGSTSGNGSGDIFIAFSTANREAARERA
Gene_236976 261 ..........ANDELDKPTRDGSIIIIVATDAPLLPHQLRRLAKRPALGLGRLGGISSDGSGDIFLAFSTASPGLINENE
Gene_241831 240 .....................GSIIVVVATDAPLLPHQLKRLARRVPLGIAKMGGNGENTSGDIFIAFSSANPESSKRVG
Gene_281166 244 .....................GSIIAVVATDAPLMPHQLKRLARRVGLGVGRSGSIAHHGSGDIFLAFSTANRAAWARGS
Gene_366662 218 ....G.EGSIIVIVATNAPLIPAQCKRLAQRATVGLARVGGVGHNGSGDIFLAFATGNHLPFNSSA
Gene_454500 279 ...................SDGSIIVVVATDAPLLPHQLKRIAQRVPLGIGIVGGRGSNGSGDIFIAFSTANKNAFDRSD
Gene_454672 269 ..................AGRGSIIIIVATDAPLLPHQLKRIARRASLGVARTGGTAGNGSGDIFVAFSTANAESAGGRP
Gene_464923 287 ESAHTREAVEAREAEDIETDRGSIIIVVATDAPLLSHQLKRIARRASLGVARTGGTAGNGSGDIFVAFSTANPGAASARP
Gene_520036 241 ..................EGDGSIIIVIATDAPLLPYQLKRLAKRAGMGLARMGSVANNGSGDIFIAFTTADQSLGRVDR
Gene_619370 241 ..................EGDGSIIIVIATDAPLLPYQLKRLAKRAGMGLARMGSVANNGSGDIFIAFTTADQSLGRVDR
Gene_673701 221 ....R.........DERGSIIVVVATDAPLLPHQLKRLARRASLGVARTGGVSGNGSGDIFIAFSNANPGTAGAEQ
Gene_738131 265 ...................DLGSIIVVVATDAPLLPHQLKRLARRVPMGIGRVGGYAGNGSGDIFIAFSTANEGAADRRE
Gene_784591 244 ..................FAAGSIIVVVATDAPLLPHQLKRLLRRAALGLARNGSIASNGSGDIFIAFSTANKDADQAGA
Gene_1065070 241 ....................QGSIIVVLGTDAPMLPHQLQRVARRAAIGVGRGGSPGGNNSGDIFLAFSVANRMPMMQMA
Gene_1141084 256 ..................DEKGSIIVVIATDAPLLPHQLKRVATRAGLGIARVGGMASNGSGDIFIAFSTANPRELRSGS
Gene_1221824 267 .....................GSIIVVVATDAPLLPNQLKRLARRVPIGISRMGGFASNGSGDIFIAFSTANAGAANRKE
Gene_1377453 266 .....................GSIIVVVATDAPLLPHQLKRLARRVPIGIGRVGGVGTNGSGDIFIAFSTANPDAWSRKE
Gene_1422059 286 .....................GSIIVVVATDAPLLPHQLERLARRVPLGIAKVGGYASNGSGDIFIAFSTANPGAATREG
Gene_1625963 267 ..............VDDIRELGSIIVVVATDAPLLPQQLKRIARRVPMGIGRVGGYASNGSGDIFIAFSTANVGAADRRE
Gene_1929613 251 ......................SIIVVVATDAPLLPQQLQRLAERVPLAIGRLGGLGGNGSGDIFIAFSTGNPGAWRERP
Gene_1932634 274 ..................AEAGSIIIVAATDAPLLPHQLKRIARRTSLGLARVGGIGGNGSGDIFIAFSTANKNAGQVAR
Gene_1B65
β13 η8 α9 β14 TT β15 α10
Gene_1B65 308 APFLDVEMVNDEPLDTVYLAAVDSVEEAVVNAMIAAEDMGGTPFDRLLVQAIDHERLRAVLRQYGRLA...
Gene_1241 309 N..LSVRLLGNEHVTPVFSGTVLATEEAIVNALVAARTMTGF..EGRTIEAIDHEALRAALRKYNRLDEPQ
Gene_68362 349 VT.SGVEILANGRLNPIFAATVEATEEAIVNAMVAAETMTGA..DDVTVYALPHDRLREALRKYNRLGG..
Gene_236976 331 E..STISMFPNNGLSVVFEAAVQATEEAIVNAMVAAETVVGA..SGLQVEEMPEDQLRAIFLD........
Gene_241831 299 L..KQLTMLPNDQMDPLFQATVQGTEEAIINAMLAAETMTGI..DGNTVHALPHDRLQVALKKYNRLL...
Gene_281166 303 GP.RDCRFLPNDAMDPLFAGVVEATDEAIVDSMVANETMTGR..DGVTAIALPHDRLSEILAEHNRLSKSS
Gene_366662 279 L..HDLKMLPHEQINSIFEATAEAVEESILNALTAAETTTGF..QGHTAHALPLDELKRVMAKYRPST...
Gene_454500 340 N..QSVVTLSNDRITPLFEATVQSVEEAIINAMVAAETMEGN..NGNKAYALPHGALVEILRKYNRLK...
Gene_454672 331 T..SDIQMLSNSRITPLFDATVQAVEEAIINALVAAETMVGR..DGNRAEALSHERLREILGRFNRLAG..
Gene_464923 367 V..AELTMLSNSRISPVFDATVEATEEAIINALIAAETMVGR..DGNRSEALDHDRLREILARYNRLAQ..
Gene_520036 303 L..LGHRSVPNDRMNPLFAATVQATEEAIVNALVAARDMTGD..RGHHITAIPHDELVEVLSRYRMIER.
Gene_619370 303 L..LGHRSVPNDRMNPLFAATVQATEEAIVNALVAARDMTGD..RGHHITAIPHDELVEVLSRYRMIER.
Gene_673701 284 R..AQLEALSNSRMDPLFKGTVEATEEAILNALVAAETMTGI..NDHTVTALPHRKTREVLKQYGRLSD..
Gene_738131 326 I..QTLEMYPNDRMSGLFEATAQAVEESIINAIIAGETMVGI..NGNTTYGIPHGRLQEVLERYNRLER..
Gene_784591 306 G..VTVRTLANENINPMFLATVEATEEAIINALIAGRDMRGH..RGNIVKAIEHDDLRSVLRDYNRLEETK
Gene_1065070 301 PHHLKLDFLNDEIFDDIYLAAVEAVEEAVLNAMVAAEDMTTLRPAGKICRALDHQQLVEIMRRYGRCS...
Gene_1141084 318 N..LSVSLLGNEHITPVFSGAVLATEEAIVNALVAARTMTGF..EGTTVEAIDHAALRAALRKYNRLDESQ
Gene_1221824 326 N..QNILMIPNDKMSALFEATAQATEESILNALISAETMIGK..NNSKVYELPEDRLIEVLKKYNRIK...
Gene_1377453 325 T..VDLEMLPNDAMSSLFKATAEATEESIVNAMVAAETMVGI..NGNKVYALPHDRLQETLREYNRLELPQ
Gene_1422059 345 L..KDIEMLPNDSMSRLFLATAQATEEAIVNALVAAETMVGI..NGNTVYALPQDRLVSILQKYNRIP...
Gene_1625963 333 N..QSIERLPNDRMSGLFEATAQAVEEAIINAIVAGETMVGI..NGNTTYGIPHDRLREVLEKYNRLER..
Gene_1929613 309 A..ASLDMLP...
Gene_1932634 336 G..VSVRMLANEDM...
